# Antibody Response to HERV-K and HERV-W Envelope Epitopes in Patients with Myasthenia Gravis

**DOI:** 10.3390/ijms25010446

**Published:** 2023-12-28

**Authors:** Elena Rita Simula, Ignazio Roberto Zarbo, Giannina Arru, Elia Sechi, Rossella Meloni, Giovanni Andrea Deiana, Paolo Solla, Leonardo Antonio Sechi

**Affiliations:** 1 Department of Biomedical Sciences, University of Sassari, Viale San Pietro 43b, 07100 Sassari, Italy; simulaelena@gmail.com; 2 Department of Medicine, Surgery and Pharmacy University of Sassari, Viale S. Pietro 10, 07100 Sassari, Italy; irzarbo@uniss.it (I.R.Z.); parentina@yahoo.com (G.A.); eliasechi87@gmail.com (E.S.); sardinia@hotmail.com (R.M.); gdeiana@uniss.it (G.A.D.); 3 Department of Medical Sciences and Public Health, University of Cagliari, 09124 Cagliari, Italy; 4 Struttura Complessa Microbiologia e Virologia, Azienda Ospedaliera Universitaria, 07100 Sassari, Italy

**Keywords:** MG, myasthenia gravis, autoantibodies, HERV-K, HERV-W, epitopes, humoral response

## Abstract

Myasthenia gravis is an antibody-mediated autoimmune neurological disorder characterized by impaired neuromuscular junction transmission, resulting in muscle weakness. Recently, the involvement of Human Endogenous Retroviruses (HERVs) in the pathophysiology of different immune-mediated and neurodegenerative diseases, such as multiple sclerosis, has been demonstrated. We aimed to investigate potential immune system involvement related to humoral responses targeting specific epitopes of HERV-K and HERV-W envelope proteins in myasthenia gravis. Myasthenia gravis patients were recruited in the Neurology Unit, while healthy controls were selected from the Blood Transfusion Center, both affiliated with AOU Sassari. Highly immunogenic antigens of HERV-K and HERV-W envelope proteins were identified using the Immune Epitope Database (IEDB) online tool. These epitopes were utilized in enzyme-linked immunosorbent assays (ELISA) to detect autoantibodies in serum directed against these sequences. The study involved 39 Healthy Donors and 47 MG patients, further categorized into subgroups based on the presence of autoantibodies: MG-AchR Ab+ (*n* = 17), MG-MuSK Ab+ (*n* = 7), double seronegative patients (MG-DSN, *n* = 18), MG-LRP4 Ab + (*n* = 4), and one patient with no antibodies data (*n* = 1). Our findings revealed high levels of autoantibodies in myasthenia gravis patients directed against the HERV-K-env-su_(19–37)_, HERV-K-env-su_(109–126)_, HERV-K-env-su_(164–186)_, HERV-W-env_(93–108)_, HERV-W-env_(129–14)_, and HERV-W-env_(248–262)_ epitopes. Notably, these results remained highly significant even when patients were subdivided into MG-AchR Ab+ and MG-DSN subgroups. Correlation analysis further revealed significant positive associations between the antibody levels against HERV-K and HERV-W families in patients, suggesting a synergistic action of the two HERVs in the pathology context since this correlation is absent in the control group. This study marks the first identification of a specific humoral response directed against defined epitopes of HERV-K and HERV-W envelope proteins in myasthenia gravis patients. These findings lay the foundation for future investigations aimed at elucidating the molecular mechanisms driving this immune response. The detection of these autoantibodies suggests the potential for novel biomarkers, especially within the MG-DSN patient subgroup, addressing the need for new biomarkers in this population.

## 1. Introduction

Human Endogenous Retroviruses (HERVs) are retroviral sequences integrated into the human genome, inherited from ancestral retroviral infections that occurred in remote forebears. They constitute a substantial fraction of our genomic architecture, accounting for approximately 8% of the human genome. A minimum of 31 distinct families of HERVs exist, each originating from a separate incursion by an exogenous viral agent [1]. The HERV-K and HERV-W families are among the most extensively studied in the field of endogenous retroviral elements in humans. Although a preponderance of these sequences remains transcriptionally dormant or functionally inactive, a subset may be transcriptionally activated and translated into functional proteins or RNAs under specific conditions. Notably, within pathological contexts characterized by autoimmune dysregulation, HERVs have been observed to undergo reactivation in response to various triggering factors, including viral infections, oxidative stress, and disruptions in immune homeostasis [2,3]. Different aspects of HERVs, including their roles in autoimmunity and gene/protein expression, have been emphasized in the context of immune-mediated diseases such as multiple sclerosis (MS) [4], neuromyelitis optica with aquaporin-4 autoantibodies [5], systemic lupus erythematosus (SLE) [6], and Sjögren’s syndrome (SjS) [7]. In fact, while proteins derived from HERVs might be designated as self-antigens due to their genomic nature, the lack of thymic expression during pivotal stages of immune tolerance establishment might render them perceptible as neoantigens [8]. Stemming from exogenous retroviral lineage, HERVs exhibit sequence homologies with their ancestral viral counterparts, potentially offering antigenic epitopes that can be recognized by lymphocytes [9]. This concept, referred to as molecular mimicry, provides insight into a potential pathway through which viral infections may precede autoimmune pathogenesis. 

In this context, evidence from our study suggests a potential involvement of HERVs in the pathogenesis of myasthenia gravis (MG), an autoimmune disorder that affects neuromuscular junction (NMJ) function. Notably, MG exhibits a higher prevalence among women compared to men, with a commonly reported gender ratio of approximately 2:1. Within this framework, acetylcholine (Ach) plays a key role in transmitting signals from motor neurons to skeletal muscle. The NMJ complex is regulated by key proteins, including acetylcholine receptors (AChRs), and Muscle-Specific Kinase (MuSK). These proteins govern receptor clustering and ion channel activation, orchestrating the essential downstream signaling cascade for muscle contraction. Disruptions in this finely regulated system can result in conditions like MG, where autoantibodies (AAbs) aberrantly target AChRs or associated postsynaptic molecules. These AAbs act as causative agents, inducing skeletal muscle dysfunction primarily manifested as observable muscular fatigue [10,11,12]. AAbs against AChR and MuSK are pivotal in MG diagnosis and they have been rigorously validated for their diagnostic specificity and sensitivity [13]. Not only do they function as diagnostic indicators, they also enable the stratification of pathological subgroups and the identification of variant pathogenicities within these subsets [14]. The diagnosis of MG is established through a combination of symptomatic presentations, clinical findings, and the detection of specific AAbs through targeted testing [15]. In the absence of detectable AAbs, the diagnosis is corroborated by neurophysiologic testing and a positive therapeutic response [16]. Additionally, the thymic status holds clinical significance, contributing to disease pathophysiology through mechanisms such as thymic hyperplasia or thymoma, both potentially linked to increased AAbs production against AchRs [17]. The thymus, central to immune adaptivity, may, in specific MG instances, foster T cells that erroneously target acetylcholine receptors, implicating a breakdown in immune tolerance [18]. First-line therapy typically focuses on symptomatic pharmacological measures, with acetylcholinesterase inhibition being the most effective [19]. In cases where desired therapeutic outcomes are not achieved, a shift to immunosuppressive pharmacotherapy is recommended, requiring careful dosage titration due to implications on treatment efficacy and potential side effects [20]. Thymectomy, a surgical intervention, has been rigorously scrutinized, highlighting its centrality in MG management [12,21,22,23,24]. Concurrently, incorporating low-to-moderate intensity exercise has been empirically shown to confer both immediate and prolonged benefits for MG patients [25]. 

In our study, we aimed to focus on the potential role of the development of autoantibodies targeting specific epitopes of HERV-K and HERV-W, with particular emphasis on the MG-DSN subgroup, which presents a serious gap in MG diagnosis and understanding.

## 2. Results

The humoral response to selected epitopes from HERV-K-env-su and HERV-W-env was examined in the plasma of the entire MG cohort and the HC group. A Mann–Whitney U test was performed to compare quantitative values of the antibodies’ OD among groups. Cut-off values were selected via ROC analysis, and a comparison between the percentage of positive and negative samples was performed using Fisher’s exact test. 

**Figure 1:**
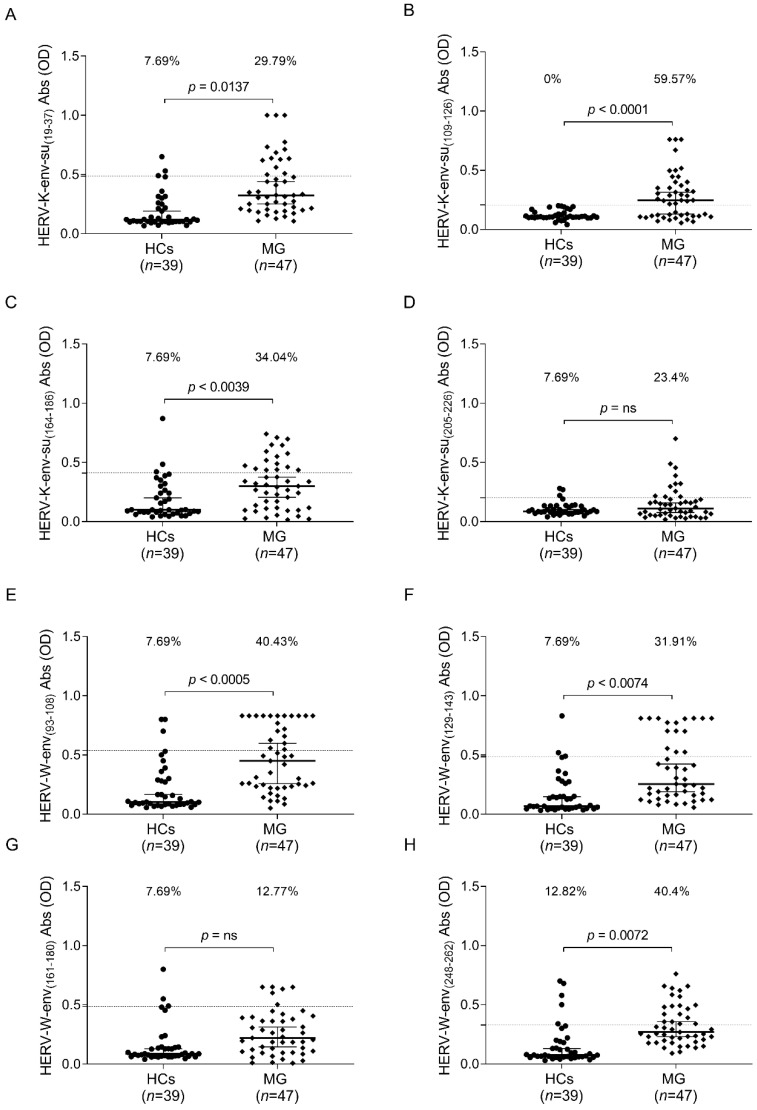
ELISA-based analysis of Abs reactivity against HERV-K-env-su- and HERV-W-env-derived peptides. Plasma samples from the entire cohort of MG patients and HCs subjects were tested against HERV-K-env-su_(19–37)_ (**A**), HERV-K-env-su_(109–126)_ (**B**), HERV-K-env-su_(164–186)_ (**C**), HERV-K-env-su_(205–226)_ (**D**), HERV-W-env_(93–108)_ (**E**), HERV-W-env_(129–143)_ (**F**), HERV-W-env_(161–180)_ (**G**), and HERV-W-env_(248–262)_ (**H**) peptides. The median and dashed lines delineate the thresholds employed to determine sample positivity. The upper section of each graph displays the *p*-value and the proportion of positive patients, as determined with Fisher’s exact test, “ns” indicates a non-significant result with a *p*-value greater than 0.05, suggesting no statistically significant differences between the compared groups.

In the comprehensive analysis (illustrated in Figure 1), we observed a statistically significant presence of AAbs against HERV-K and HERV-W in the MG patient group when compared to the control group, except for the epitopes HERV-K-env-su_(205–226)_ (Figure 1D) and HERV-K-env-su_(161–180)_ (Figure 1G). 

Notably, AAbs targeting the epitope HERV-K-env-su_(19–37)_ were detected in 30% of MG patients compared to 8% of HCs, as corroborated by Fisher’s exact test (*p* = 0.0137) (Figure 1A). Regarding HERV-K-env-su_(109–126)_, the assay allowed us to differentiate between patient and control populations with a specificity of 100%. A total of 60% of MG patients exhibited a humoral response against this peptide, while none within the control group demonstrated reactivity towards the same epitope (Figure 1B). 

For the final immunogenic epitope of HERV-K studied in our MG cohort, HERV-K-env-su_(164–186)_, we detected the presence of AAbs in 34% of MG patients in contrast to 8% in the control group, with a *p*-value of 0.0039 (Fisher’s exact test) (Figure 1C). Concerning the humoral response against HERV-W, we observed that over 30% of MG patients displayed humoral reactivity toward the epitopes HERV-W-env-su_(93–108)_ and HERV-W-env-su_(129–143)_ (Fisher’s exact test: *p* = 0.0005 and *p* = 0.0074, respectively). In contrast, a relatively small fraction of the HCs, slightly above 7%, exhibited AAbs reactivity to the same epitopes (Figure 1E,F). For the epitope HERV-K-env-su_(248–262)_, which emerged as the most immunogenic in our study, specific AAbs were detected in 62% of the MG patient population, in contrast to 18% in the healthy control group (Figure 1H). 

As part of the subclassification based on the presence of AAbs among MG patients, we conducted a detailed analysis to identify specific AAbs in MG subgroups categorized as MG AChR Ab+, MG MuSK Ab+, and MG Double Seronegative (MG-DSN). The subgroups, which include four patients who tested positive for LRP4 Abs and one subject with missing Abs data, are too small to provide reliable results in statistical analyses. Therefore, these subgroups were not considered in the detailed analyses discussed in the subsequent sections of this paper. 

**Figure 2:**
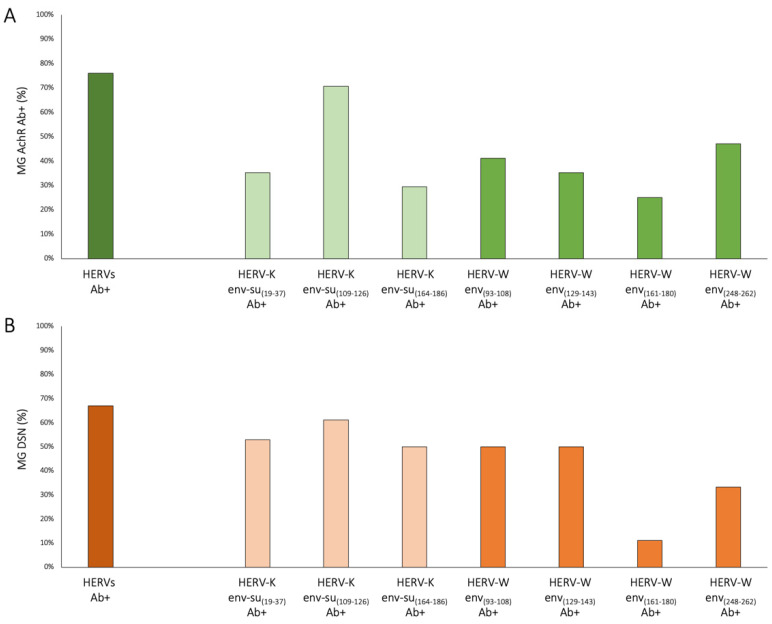
Summary graph depicting the positivity rates among MG patients, categorized into AchR Ab+ (**A**) and DSN (**B**), in response to peptides derived from HERVs. The first bar, in deep green (**A**)/orange (**B**), represents the overall percentage of patients who tested positive for at least one of the epitopes under investigation. The bars located on the right side of the graph display the patient positivity rates for each specific peptide: three bars in light green (**A**)/orange (**B**) for HERV-K peptides, and the four bars in a darker shade of green (**A**)/orange (**B**) for HERV-W peptides.

Figure 2 explains the humoral response of MG patients to peptides derived from HERVs. In panel A, the graph illustrates the percentage of MG-AchR Ab+ patients who exhibited positive responses to targeted HERV-envelope peptides. Panel B presents the percentages of MG-DSN patients who manifested a specific humoral response to HERV epitopes. The yellow bar in the graphs represent the percentage of patients who tested positive for at least one peptide of HERVs, whereas the right portion of the graph displays the patient positivity percentages for each peptide. It is noteworthy that a significant number of patients exhibited positive responses to multiple epitopes. The analysis excluded the HERV-K-env_(205–226)_ epitope due to the absence of AAbs in patients. Additionally, the MG-Musk-Ab+ subgroup was omitted from the summary graph, as no patients showed reactivity towards the peptides under investigation. 

**Figure 3:**
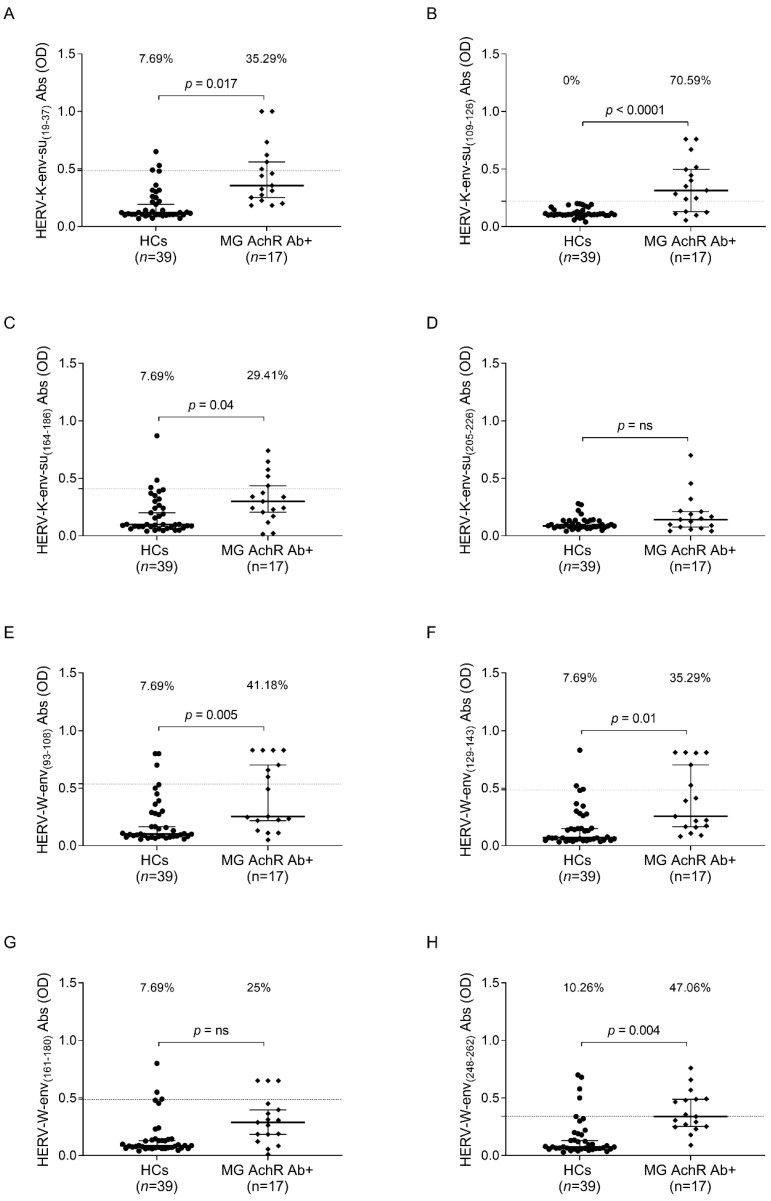
ELISA-based analysis of Abs reactivity against HERV-K-env-su- and HERV-W-env-derived epitopes. Plasma samples from MG AchR Ab+ patients and HCs subjects were tested against HERV-K-env-su_(19–37)_ (**A**), HERV-K-env-su_(109–126)_ (**B**), HERV-K-env-su_(164–186)_ (**C**), HERV-K-env-su_(205–226)_ (**D**), HERV-W-env_(93–108)_ (**E**), HERV-W-env_(129–143)_ (**F**), HERV-W-env_(161–180)_ (**G**), and HERV-W-env_(248–262)_ (**H**) peptides. The median and dashed lines delineate the thresholds employed to determine sample positivity. The upper section of each graph displays the *p*-value and the proportion of positive patients, as determined with Fisher’s exact test, “ns” indicates a non-significant result with a *p*-value greater than 0.05, suggesting no statistically significant differences between the compared groups.

In the following analysis, the HCs population was compared with the MG patient subgroups. We observed a heightened humoral response in the MG AchR Ab+ subgroup compared to the controls for the peptides HERV-K-env-su_(19–37)_, with a *p*-value of 0.017 (Figure 3A), and HERV-K-env-su_(109–126)_, which showed 100% specificity, identifying 70.59% of the patients as positive for AAbs (Figure 3B). Additionally, for HERV-K-env-su_(164–186)_, a *p*-value of 0.005 was observed (Figure 3C), and for HERV-W-env_(93–108)_, 41.18% of the patients were positive compared to 7.69% of the controls (Figure 3E). Regarding HERV-W-env_(129–143)_ (Figure 3F) and HERV-W-env_(248–262)_ (Figure 3H), more than 35% of patients exhibited peptide positivity. Conversely, no significant differences were observed between the patient and control populations for the epitopes HERV-K-env-su_(205–226)_ and HERV-W-env_(161–180)_ (Figure 3D,G).

**Figure 4:**
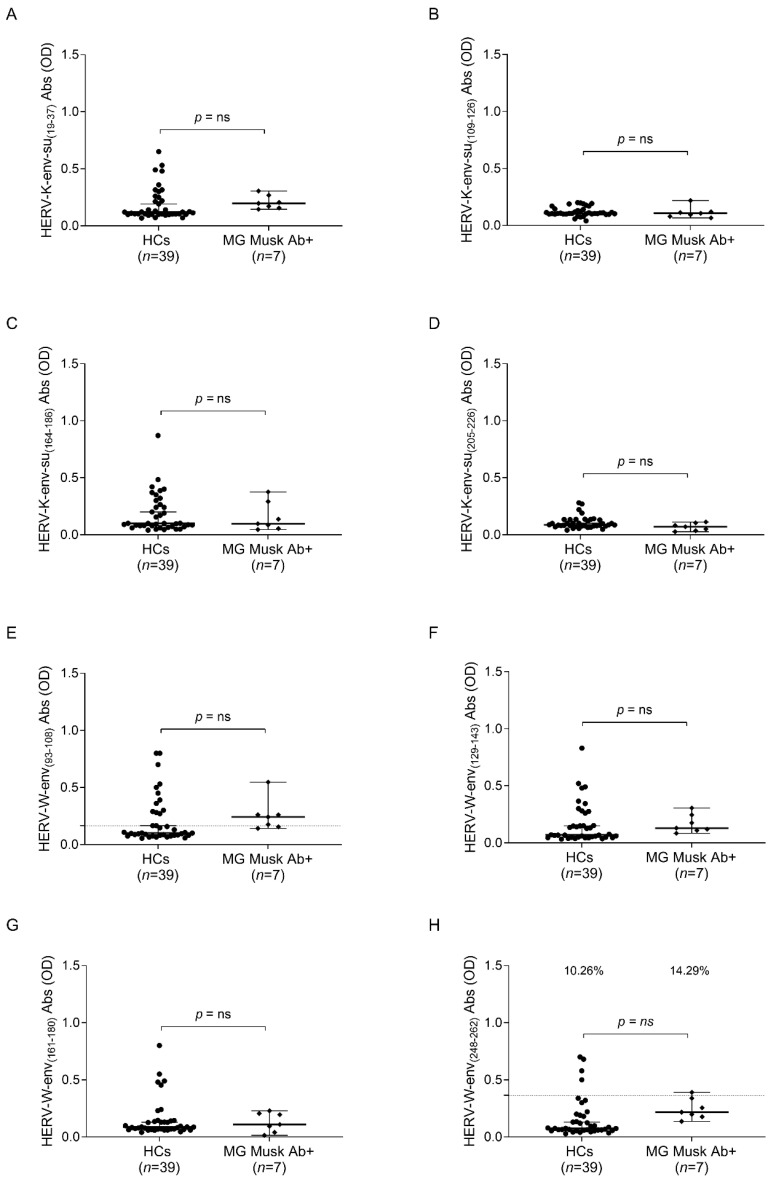
ELISA-based analysis of Abs reactivity against HERV-K-env-su and HERV-W-env-su derived peptides. Plasma samples from MG Musk Ab+ patients and HCs subjects were tested against HERV-K-env-su_(19–37)_ (**A**), HERV-K-env-su_(109–126)_ (**B**), HERV-K-env-su_(164–186)_ (**C**), HERV-K-env-su_(205–226)_ (**D**), HERV-W-env_(93–108)_ (**E**), HERV-W-env_(129–143)_ (**F**), HERV-W-env_(161–180)_ (**G**), and HERV-W-env_(248–262)_ (**H**) peptides. The median and dashed lines delineate the thresholds employed to determine sample positivity. The upper section of each graph displays the *p*-value and the proportion of positive patients, as determined with Fisher’s exact test, “ns” indicates a non-significant result with a *p*-value greater than 0.05, suggesting no statistically significant differences between the compared groups.

There were no notable differences in humoral responses within the MG Musk Ab subgroup compared to healthy controls. However, this analysis may be heavily influenced by the small number of subjects in the patient population (Figure 4).

**Figure 5:**
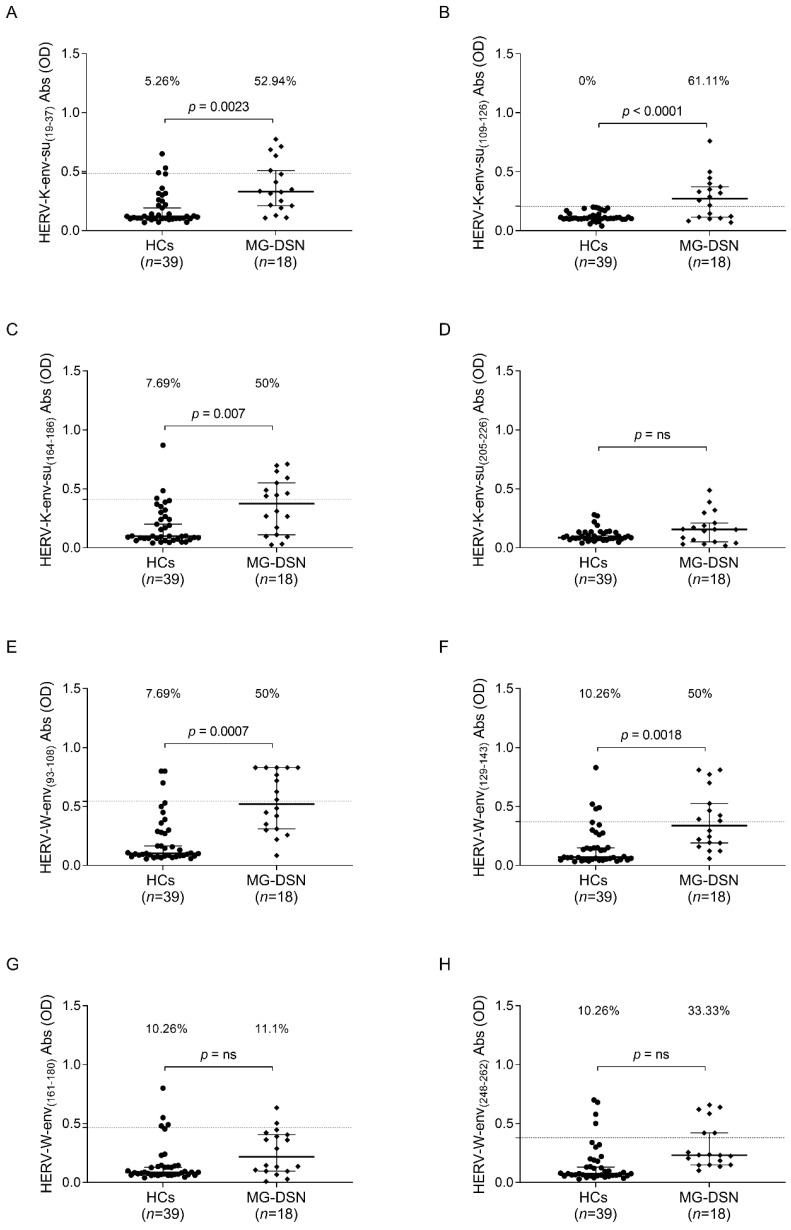
ELISA-based analysis of Abs reactivity against HERV-K-env-su- and HERV-W-env-su-derived peptides. Plasma samples from MG-DSN patients and HCs subjects were tested against HERV-K-env-su_(19–37)_ (**A**), HERV-K-env-su_(109–126)_ (**B**), HERV-K-env-su_(164–186)_ (**C**), HERV-K-env-su_(205–226)_ (**D**), HERV-W-env_(93–108)_ (**E**), HERV-W-env_(129–143)_ (**F**), HERV-W-env_(161–180)_ (**G**), and HERV-W-env_(248–262)_ (**H**) peptides. The median and dashed lines delineate the thresholds employed to determine sample positivity. The upper section of each graph displays the *p*-value and the proportion of positive patients, as determined with Fisher’s exact test, “ns” indicates a non-significant result with a *p*-value greater than 0.05, suggesting no statistically significant differences between the compared groups.

The investigation into the MG-DSN subgroup yielded particularly intriguing insights. Notably, for this patient cluster, no established AAbs currently serve as diagnostic biomarkers. Significant differences were observed in the response to selected epitopes in the MG-DSN group. Specifically, for peptides HERV-K-env-su_(19–37)_ (*p* = 0.0023, Figure 5A), HERV-K-env-su_(109–126)_ (*p* < 0.0001, Figure 5B), HERV-K-env-su_(164–186)_ (*p* = 0.0007, Figure 5C), HERV-W-env_(93–108)_ (*p* = 0.0007, Figure 5E), and HERV-W-env_(129–143)_ (*p* = 0.0018, Figure 5F), an equal or greater than 50% proportion of patients tested positive for the sought antibodies. However, no significant differences were observed for peptides HERV-K-env-su_(205–226)_, HERV-W-env_(161–180)_, and the epitope HERV-K-env-su_(248–262)_ (Figure 5D,G,H).

To minimize bias in our study design and lessen differences between the populations, each patient was specifically matched with one control subject based on gender and age. The findings from this analysis were more significant compared to our previous analysis, as will be detailed below and in the supplementary materials. 

In the MG AChR Ab+ subgroup, our tests yielded a discriminating specificity of 100% when distinguishing between the patient and control populations for all investigated peptides, except for the peptide HERV-W-env_(161–180)_. For this specific epitope, a specificity exceeding 90% was rigorously maintained. Remarkably, 82% of the MG-AChR Ab + patients manifested AAbs against this epitope, in stark contrast to a mere 6% observed in the HCs (Figure S1G). For all other studied epitopes, none of the control subjects exhibited positive reactivity, while in the MG AChR+ subgroup, an 82% prevalence was discerned for the epitopes HERV-K-env-su_(19–37)_, HERV-K-env-su_(109–126)_, HERV-K-env-su_(164–186)_, HERV-W-env_(129–143)_, and HERV-W-env_(161–180)_ (Figure S1A–C,F,G, respectively). 

The proportion of positive patients exhibited a slight decline in the case of the epitopes HERV-K-env-su_(205–226)_ and HERV-W-env_(93–108)_, where 59% and 76% of MG-AChR+ patients were found to be positive for these respective epitopes (Figure S1D,E). 

In the case of the epitope HERV-W-env_(248–262)_, an increase in the proportion of positively reacting patients was noted, reaching as high as 94% (Figure S1H). 

A comparable analysis was conducted within the MG MuskAb+ patient subgroup. This examination revealed no significant differences between the patient cohort and the control group. However, in the context of two HERV-W epitopes, HERV-W-env_(93–108)_ and HERV-W-env_(248–262)_, a significant divergence was observed, with 71% and 100% of the patients testing positive, respectively (Figure S2E,H). 

Regarding the MG-DSN subgroup, our empirical examination achieved a specificity of 100% for six out of the eight epitopes under investigation when distinguishing between patients and controls. AAbs against the epitope HERV-K-env-su_(109–126)_ were detected in 59% of the patients within this subgroup (Figure S3B), while those against the epitope HERV-K-env-su_(164–186)_ were detectable in 65% of the cohort (Figure S3C). 

Within the envelope domain of HERV-W, we observed a pronounced humoral reactivity among 72% of the patients against the epitopes HERV-W-env_(129–143)_ and HERV-W-env_(161–180)_ (Figure S3F,G). Interestingly, the two most immunogenic epitopes, HERV-W-env_(93–108)_ and HERV-W-env_(248–262)_, were recognized in 94% and 100% of the patient population, respectively (Figure S3E,H). The unique outlier in our study was the epitope HERV-K-env-su_(19–37)_, for which the assay did not provide an unequivocal 100% discrimination between patients and controls. Nonetheless, the test still offered a specificity exceeding 90%, with 53% of patients showing a positive response to the peptide and a mere 5% reactivity among the control group (Figure S3A). 

**Figure 6:**
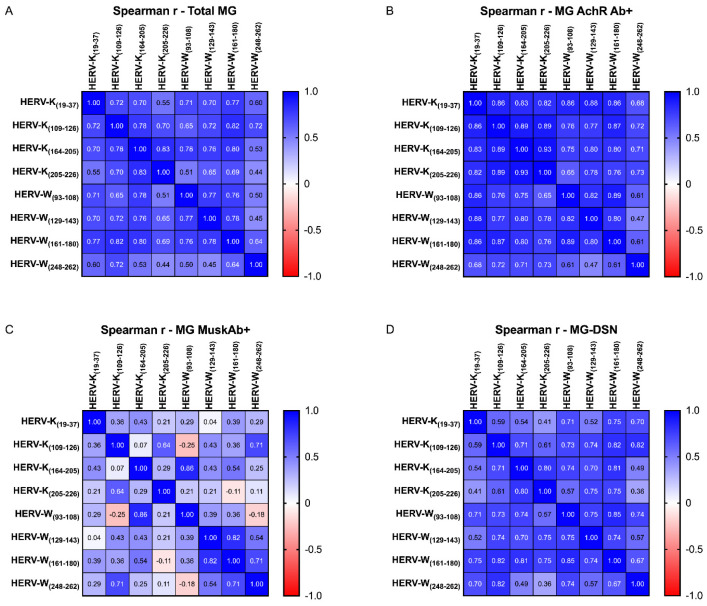
Heatmap displaying the r values obtained from Spearman correlation analysis performed among HERV-K- and HERV-W-derived epitopes in the entire MG cohort (**A**), MG AchR Ab+ (**B**) subjects, MG Musk Ab+ cohort (**C**), and MG-DNS patients (**D**).

In the final phase of our investigation, we endeavored to ascertain the presence or absence of a correlation between AAbs directed against epitopes of HERV-K and those targeting HERV-W. The results of our analysis revealed the presence of a positive correlation, as illustrated in Figure 6, exclusively within the patient population and not among the control subjects. This observation leads to the hypothesis that there may be an underlying mechanism that can be exploited to trigger the reactivation of HERVs, which subsequently operate synergistically, specifically within the pathological context, as opposed to physiological conditions. 

## 3. Discussion

The potential involvement of HERVs in neurological conditions [26,27,28] and autoimmune disorders [29] has been extensively documented. 

Different studies revealed the presence of antibodies against retroviral proteins in the sera of patients with diverse autoimmune disorders [30,31]. The transcriptional activation of a subset of HERVs in RA patients, particularly the HERV-K family, has been observed [32]. Subsequent research identified that approximately 19% of RA patients harbored Abs against specific epitopes within the HERV-K envelope, HERV-K-env_(19–37)_ [33]. 

In Type 1 Diabetes Mellitus (T1D), a notable antibody response to HERV-K-env_(19−37)_ was significantly increased in patients when compared to HCs [34]. 

In addition, the same peptides employed in the current study, HERV-W-env_(93–108)_ and HERV-W-env_(248–262)_, showed significant immunoreactivity with recognition rates of 31.25% (*p* < 0.0001) in MS patients and 15% (*p* = 0.02) in the HCs group [4].

Reactivation of HERVs is not exclusively associated with autoimmune diseases; it can also be prompted by the presence of viral agents. It has been observed that Epstein–Barr virus (EBV) induces transcriptional activation of the env gene of HERV-K18, which exhibits superantigen (SAg) activity [35]. 

Recent research has revealed that the transcriptome of HERVs undergoes dynamic modulation during SARS-CoV-2 infection, and this modulation enables the distinct differentiation of various clinical stages of COVID-19 [36]. 

In our investigation, we focused on elucidating the involvement of HERVs in the immune system, with specific emphasis on MG. 

The hypothesis that HERVs may play a central role and intricately interact with our immune system is currently a dynamic and burgeoning field of scientific inquiry. As previously highlighted in the introduction, proteins originating from HERVs may be classified as self-antigens [37] or even recognized as neoantigens [8]. Proteins of both viral and bacterial origin, in conjunction with autologous proteins, exhibit structural, functional, or immunological similarities. Within this intricate landscape, a putative sequence homology identified between the envelope proteins of the HERV-W family and myelin is proposed to act as a potential trigger for the immunological cascade observed in MS [38]. This phenomenon, known as molecular mimicry, raises intriguing questions about immunopathological pathways in which sequence homology could serve as a channel for autoimmunity. Due to the deep structural and functional congruence between specific proteins, an immune response directed toward retroviral epitopes might unintentionally generate cross-reactivity, thereby triggering a sequence of autoimmune events [38,39]. Our scientific inquiry has been particularly focused on the envelope protein, primarily because retroviral envelope proteins are theorized to wield the dual capacity to both initiate and suppress immune reactions. Within this realm, a peptide comprised of 14 amino acids (LQARILAVERYLKD), nestled within the transmembrane glycoprotein (TM) gp41 of HIV-1, obstructs the lymphokine-dependent proliferation of T lymphocytes incited by mitogens. Sequences bearing resemblance to the aforementioned peptide, although not exact replicas, have been identified within several HERV lineages, including HERV-W, HERV-FRD, and HERV-K [40]. Notably, this peptide also plays a role in modulating cytokine profiles, leading to increased concentrations of interleukin-6 (IL-6) and interleukin-10 (IL-10), while simultaneously reducing the expression of interleukin-2 (IL-2) and chemokine (C-X-C motif) ligand 9 (CXCL9) in human peripheral blood mononuclear cells (PBMCs). Such modulation aids in the perpetuation and amplification of viral activities within host cells. 

In our investigation, the interplay of HERVs within the immune system becomes strikingly evident. Our observations revealed a significant humoral response targeting immunogenic epitopes derived from the envelope sequences of the two most studied HERVs, HERV-K and HERV-W. This pronounced response was more significant in MG patients than in their healthy counterparts. This discrepancy was tangible in various subgroups of MG, except for the Musk Ab+ subgroup, a deviation potentially attributable to the limited number of subjects. 

MG cases that are notably absent of discernible AAbs against pivotal targets such as AChR, MuSK, or LRP4 (MG-DSN) hold allure in the scientific realm and encompass a cohort with distinct pathogenic heterogeneity. Interestingly, for certain patients within this subset, there exist AAbs, either with low affinity or at sub-threshold concentrations, directed against AChR, MuSK, or antigenic constituents of LRP4. These elusive AAbs may escape detection by traditional screening paradigms, rendering their identification feasible predominantly through cell-based assays. Demonstrating pathogenic capacity in vivo, low-affinity AAbs induce pathological manifestations similar to those observed in MG cases in which AAbs are openly identifiable. Intriguingly, it is estimated that such low-affinity AAbs characterize between 20–50% of the generalized MG population devoid of detectable AAbs [41,42]. Concurrently, AAbs with specificity towards agrin frequently coexist with varied AAbs spectra [42,43,44], although their symbiotic interactions with alternate target proteins remain shrouded in partial ambiguity. As diagnosis becomes intricate and multifaceted in scenarios without definitive AAbs, it becomes imperative to broaden the scope of the investigation to encompass myasthenic syndromes not associated with MG, as well as a multitude of conditions with both muscular and extra-muscular origins [12]. In the gap created by the absence of recognizable AAbs, clinicians must remain vigilant about the spectrum of myasthenic syndromes unrelated to MG and conditions that manifest with comparable clinical presentations, albeit without autoimmune etiology. Such conditions could be related to aberrations in neuromuscular communication or result from intrinsic muscle abnormalities. The diagnostic perspective must also include muscle pathologies, ranging from hereditary to acquired myopathies, which precipitate muscle debilitation and subsequent impairment of contractile function. Additionally, it becomes critical to recognize non-muscular pathologies, including neurological conditions that affect central or peripheral neural architectures, as well as systemic diseases that exert disruptive effects on muscle dynamics. 

Within this intricate diagnostic scenario, our investigation emerges as a relevant observation, facilitating differentiation between patients and controls in a field where numerous assessments and validations remain imperative. Our hypothesis is that increased gene expression within the envelopes of HERV-K and HERV-W may catalyze an unbalanced immune response toward these retroviruses. This, in turn, could be entangled with the pathophysiology of MG, a path we endeavor to explore further in the near future. 

However, our study presents some limitations. The size of the studied population is relatively small, prompting us to validate our findings with a larger population. Our study to date has not incorporated an examination of the correlation between Ab levels and the clinical profiles of the patients. We are actively engaged in the acquisition of clinical data to enable a more comprehensive analysis. Furthermore, although our data emphasize the involvement of HERVs in the immune response, the current scope of our experiments does not extend to delineating the precise molecular mechanisms that could be targeted for therapeutic interventions. The intricate involvement of HERVs in the immune system, particularly within the framework of MG, remains an area requiring deeper exploration. Additional research is essential to unravel the complex interactions of HERVs in immune regulation and to potentially identify novel therapeutic targets. This understanding could pave the way for innovative treatment strategies, especially in conditions like DSN-MG.

However, the main significance of these findings is their potential impact on future research. Upcoming longitudinal studies could help determine if AAbs levels change over time and relate to symptom severity. We plan to integrate additional control groups, encompassing patients diagnosed with dermatomyositis, Lambert–Eaton syndrome, and rheumatoid arthritis. This is undertaken to determine whether the presence of autoantibodies against HERV-K and HERV-W is specific to patients with MG. Additionally, in vitro tests or animal models may clarify how these AAbs influence disease progression. The discovery of AAbs targeting the envelope protein epitopes of HERV-K and HERV-W in MG patients leads to many questions about the underlying molecular interactions. 

## 4. Materials and Methods

### 4.1. Samples Collection

Samples from 47 MG patients (27 females and 20 males; median age = 56 years) were collected. To establish a suitable control group, we recruited 39 age- and gender-matched healthy individuals (HCs) from the Blood Transfusion Centre of Sassari (22 females and 17 males; median age = 58 years). The cohort was further categorized into subgroups based on the type of MG, as detailed in Table 1. 

The MG patient population was composed of individuals who tested positive for Anti-Acetylcholine Receptor Antibody (AchR Ab+) (*n* = 17, female% = 59; median age 58 years), subjects who tested positive for anti-Muscle-Specific Kinase Antibody (Musk Ab+) (*n* = 7, 71% female; median age 59 years), and individuals with Seronegative Myasthenia Gravis (DSN) (*n* = 18, female% = 44; median age 50.5 years), hospitalized at the Neurology Unit of Sassari with stored serum samples available. 

Peripheral venous blood samples were collected into K2-EDTA tubes. The whole blood was then delicately layered over an equivalent volume of Ficoll (Sigma-Aldrich, St. Louis, MO, USA) within a 15 mL centrifuge tube and spun at 1800 RPM for 20 min without brake. Plasma, located in the uppermost layer, was subsequently pipetted and analyzed through ELISA assay for the presence of AAbs targeting epitopes derived from HERV-Kenv-su and HERV-W-env. Prior to their participation in the study, all subjects provided informed consent. The study was conducted in accordance with the Declaration of Helsinki, and the protocol was approved by the Institutional Review Board ASL 1 Prot 2150/CE.

### 4.2. Peptides

The epitopes HERV-K-env-su_(19–37)_, HERV-K-env-su_(109–126)_, HERV-K-env-su_(164–186)_, HERV-K-env-su_(205–226)_ HERV-W-env_(93–108)_, HERV-W-env_(129–143)_, HERV-W-env_(161–180)_, and HERV-W-env_(248–262)_, derived from the HERVs surface protein envelope, were identified using the IEDB software (https://www.iedb.org (accessed on 26 December 2023)) that identifies potential protein regions anticipated to be recognized as B-cell epitopes. The epitopes were synthesized at >95% purity, 4mg, by Life Tein company (Life Tein, South Plainfield, NJ 07080, USA). All peptides were resuspended in DMSO and stored at −80 °C in single-use aliquots for a final concentration of 10 mM (Table 2).

### 4.3. Enzyme-Linked Immunosorbent Assay (ELISA)

Indirect ELISA was performed to detect specific Abs against HERV-K-env-su_(19–37)_, HERV-K-env-su_(109–126)_, HERV-K-env-su_(164–186)_, HERV-K-env-su_(205–226)_, HERV-W-env_(93–108)_, HERV-W-env_(129–143)_, HERV-W-env_(161–180)_, and HERV-W-env_(248–262)_ epitopes. Ninety-six-well Nunc immuno-plates were incubated overnight at 4◦C with a solution 0.05 M of carbonate—bicarbonate, pH 9.5 (Sigma-Aldrich, St. Louis, MO, USA), and the respective peptides at 10 μg/mL, for a final volume of 50 μL/well. The plates were incubated for 1h at room temperature (RT) in a blocking solution with 5% non-fat dried milk (Sigma-Aldrich, St. Louis, MO, USA) and phosphate-buffered saline (PBS), with a final volume of 200 μL/well, and washed twice in a solution with 0.05% Tween-20 and PBS (PBS-T). The plasma samples were added at 1:100 dilution and incubated for 2 h at RT. After this, each plate was washed 5 times in PBS-T and incubated for 1 h at RT with 100 μL of PBS and alkaline phosphate-conjugated goat anti-human IgG polyclonal antibody (1:1000, Sigma-Aldrich, St. Louis, MO, USA). After another washing step in PBS-T, plates were incubated in a dark environment for 8 to 10 m in milli-Q water and p-nitrophenyl phosphate following the manufacturer’s instructions (Sigma-Aldrich, St. Louis, MO, USA), and an absorbance of 405 nm was recorded using a SpectraMax Plus 384 microplate reader (Molecular Devices, Sunnyvale, CA, USA). To minimize potential variables that might influence the results, each sample was run in duplicate, and normalization was performed with the same positive control used in all plates. The positive control consisted of a plasma sample, identified at the beginning of the experimental phase, that consistently showed reactivity toward the peptide of interest. Background activity was calculated as the mean signal of an immobilized peptide with secondary Ab. Results are expressed as the means of duplicates at 405 nm optical density (OD) values. 

### 4.4. Statistical Analysis

All data were analyzed using GraphPad Prism 8.2.0 software (GraphPad Software, San Diego, CA, USA). Non-parametric continuous variables were analyzed using the Mann–Whitney U test to discern differences between the two groups. The optimal cut-off value for sample positivity was determined using the receiver-operating characteristic (ROC) curve, aiming for a specificity greater than 85%. Based on the selected cut-off, samples were classified as positive or negative and evaluated using Fisher’s exact test. 

A significance threshold was established at *p* < 0.05. Correlations between levels of AAbs against HERV-K-env-su- and HERV-W-env-derived epitopes were assessed with the Spearman correlation test.

## Figures and Tables

**Table 1:** Details on study populations.

Study Population		Number of Subjects	Gender (% Female)	Median Age
Healthy controls		39	56.41	58
	MG-AchRAb+	17	58.82	58
Myasthenia Gravis	MG-MuskAb+	7	71.43	59
	MG-DSN	18	44.44	50.5
	MG-LRP4Ab+	4	25	62.5
	No Abs data	1	100	18

**Table 2:** Details on epitopes identified in HERV-K and HERV-W envelope proteins.

Epitope Name	Epitope Position	Epitope Sequence
HERV-K-env-su_(19–37)_	Envelope-surface aa. 19–37	VWVPGPTDDRCPAKPEEEG
HERV-K-env-su_(109–126)_	Envelope-surface aa. 109–126	RPKGKTCPKEIPKGSKNT
HERV-K-env-su_(164–186)_	Envelope-surface aa. 164–186	SGQTQSCPSAQVSPAVDSDLTES
HERV-K-env-su_(205–226)_	Envelope-surface aa. 205–226	EKGISTPRPKIISPVSGPEHPE
HERV-W-env_(93–108)_	Envelope aa. 93–108	NPSCPGGLGVTVCWTY
HERV-W-env_(129–143)_	Envelope aa. 129–143	VKEVISQLTRVHGTS
HERV-W-env_(161–180)_	Envelope aa. 161–180	HTRLVSLFNTTLTGLHEVSA
HERV-W-env_(248–262)_	Envelope aa. 248–262	NSQCIRWVTPPTQIV

## Data Availability

Data is contained within the article and the supplementary material.

## Supplementary Materials

The supporting information can be downloaded at: https://www.mdpi.com/article/10.3390/ijms25010446/s1.
