# Supplementary material for: Antibody Response to HERV-K and HERV-W Envelope Epitopes in Patients with Myasthenia Gravis
Source: Int J Mol Sci. 2023 Dec 28;25(1):446. doi: 10.3390/ijms25010446 (PMC10778599; doi:10.3390/ijms25010446)
Supplement: Supplementary file 1 [file ijms-25-00446-s001.zip › ijms-2764287-supplementary.pdf]

Supplementary material

Analysis of humoral response in MG subgroups categorized as MG AChR Ab+, MG MuSK Ab+, and MG Double Seronegative (MG-DSN). Each patient was specifically matched with one control subject based on gender and age.

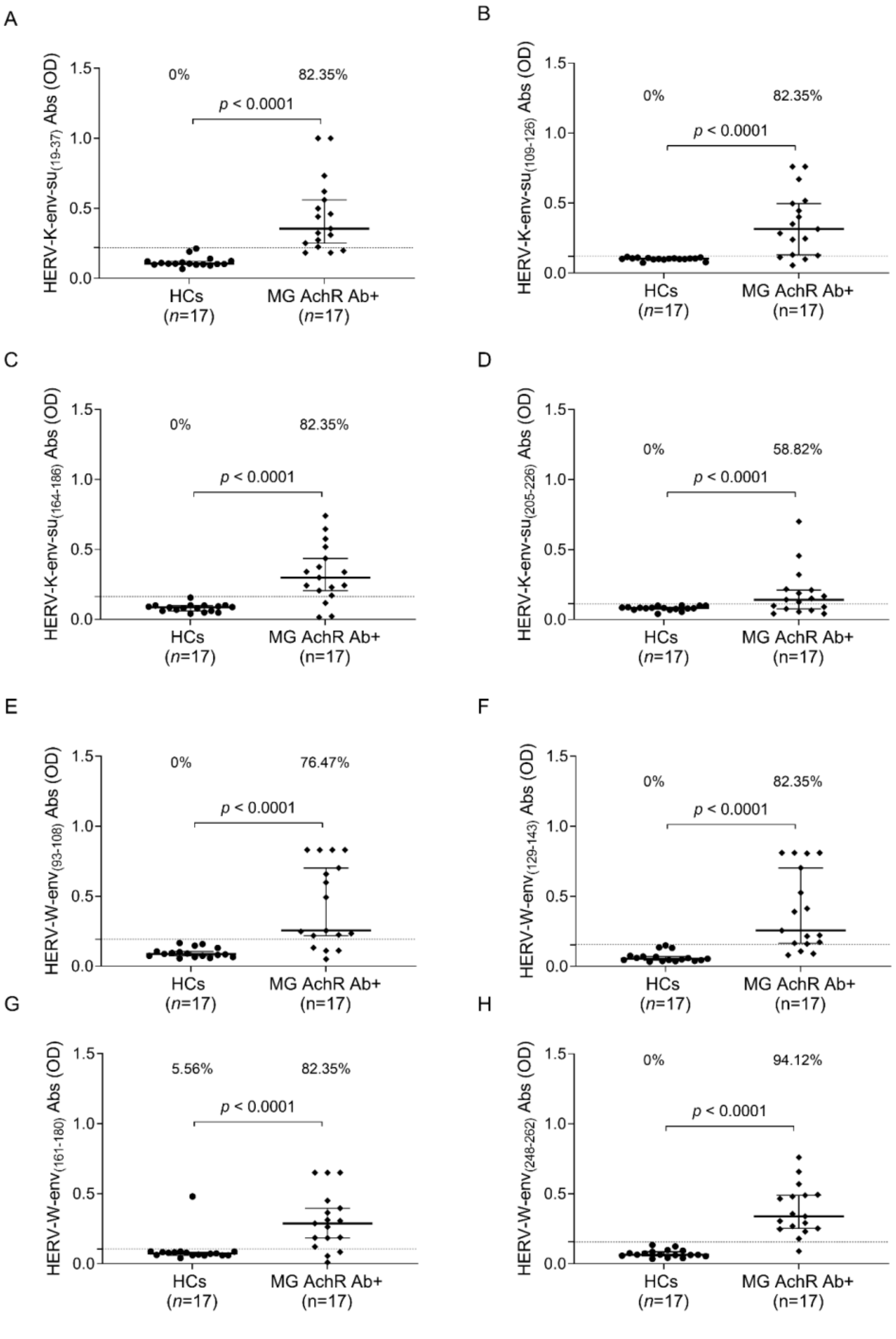

Figure S1. ELISA-based analysis of Abs reactivity against HERV-K-env-su and HERV-W-env-su derived peptides. Plasma samples from the MG-AchR Ab+ patients and HCs subjects were tested against HERV-K-env-su$_{(19-37)}$(A), HERV-K-env-su$_{(109-126)}$ (B), HERV-K-env-su$_{(164-186)}$ (C), HERV-K-env-su$_{(205-226)}$ (D), HERV-W-env-su$_{(93-108)}$ (E), HERV-K-env-su$_{(129-143)}$ (F), HERV-K-env-su$_{(161-180)}$ (G) and HERV-K-env-su$_{(248-262)}$ (H) peptides. The median and dashed lines delineate the thresholds employed to determine sample positivity. The upper section of each graph displays the *p*-value and the proportion of positive patients, as determined by Fisher's exact test.

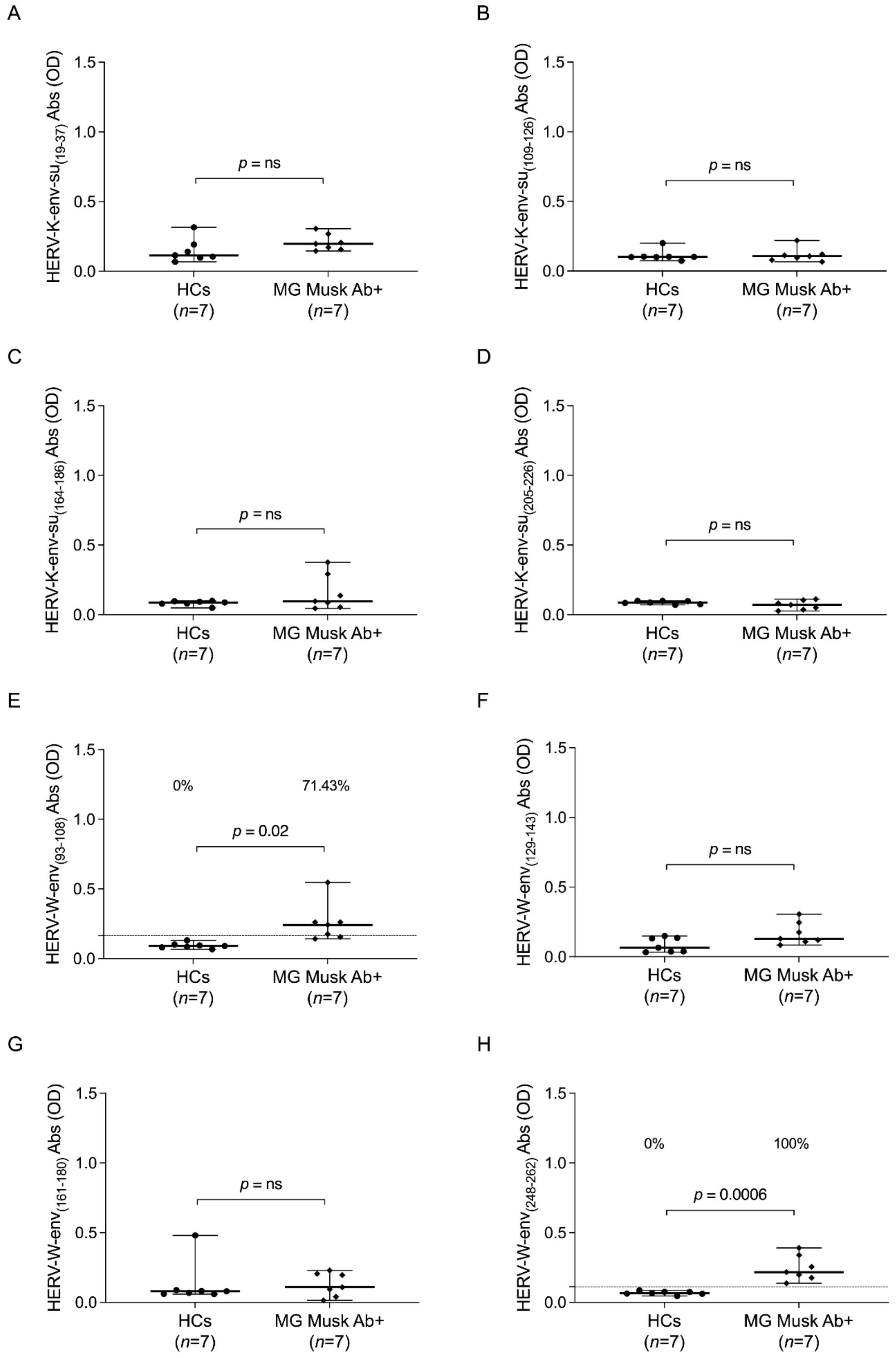

Figure S2. ELISA-based analysis of Abs reactivity against HERV-K-env-su and HERV-W-env-su derived peptides. Plasma samples from the MG-Musk Ab+ patients and HCs subjects were tested against HERV-K-env-su$_{(19-37)}$(A), HERV-K-env-su$_{(109-126)}$ (B), HERV-K-env-su$_{(164-186)}$ (C), HERV-K-env-su$_{(205-226)}$ (D), HERV-W-env-su$_{(93-108)}$ (E), HERV-K-env-su$_{(129-143)}$ (F), HERV-K-env-su$_{(161-180)}$ (G) and HERV-K-env-su$_{(248-262)}$ (H) peptides. The median and dashed lines delineate the thresholds employed to determine sample positivity. The upper section of each graph displays the p-value and the proportion of positive patients, as determined by Fisher's exact test.

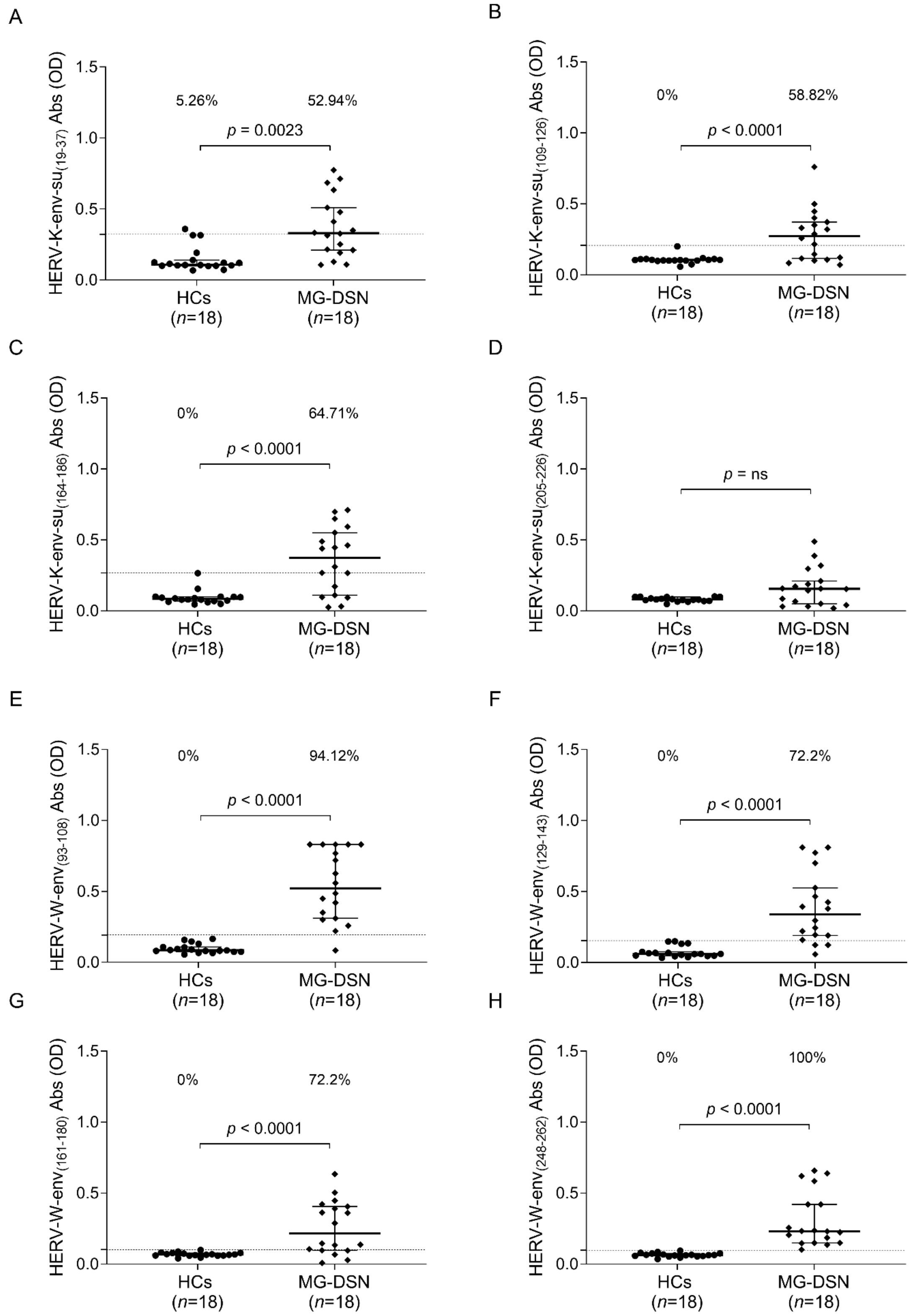

Figure S3. ELISA-based analysis of Abs reactivity against HERV-K-env-su and HERV-W-env-su derived peptides. Plasma samples from the MG-DSN patients and HCs subjects were tested against HERV-K-env-su$_{(19-37)}$(A), HERV-K-env-su$_{(109-126)}$ (B), HERV-K-env-su$_{(164-186)}$ (C), HERV-K-env-su$_{(205-226)}$ (D), HERV-W-env-su$_{(93-108)}$ (E), HERV-K-env-su$_{(129-143)}$ (F), HERV-K-env-su$_{(161-180)}$ (G) and HERV-K-env-su$_{(248-262)}$ (H) peptides. The median and dashed lines delineate the thresholds employed to determine sample positivity. The upper section of each graph displays the *p*-value and the proportion of positive patients, as determined by Fisher's exact test.
